# Language structure reflects biases in pattern learning across domains and modalities

**DOI:** 10.1177/17470218241282404

**Published:** 2024-11-14

**Authors:** Jennifer Culbertson, Arianna Compostella, Simon Kirby

**Affiliations:** 1 School of Philosophy, Psychology and Language Sciences, The University of Edinburgh, Edinburgh, UK; 2 Department of Foreign Languages and Literatures, University of Verona, Verona, Italy

**Keywords:** Language, pattern learning, cognitive biases, syntax

## Abstract

A central goal for psychological science is the explanation of variation in human behaviour. In the domain of language, patterns of cross-linguistic variation have been extensively documented, but there has been vigorous debate over how to explain them. A particularly contentious question is whether constraints on linguistic variation are driven by properties of the human mind that are specific to language or domain-general. In this paper, we present four pattern-learning experiments (*N* = 306 English- and Italian-speaking adults) across domains (linguistic and nonlinguistic) and modalities (visual, auditory, and tactile) to show that the patterns that are more easily learned are precisely the ones that are found most frequently across languages. This supports a domain-general, cognitive explanation for cross-linguistic variation. However, we suggest that the general/specific dichotomy is ultimately misleading because language structure arises when domain- and modality-general biases meet domain-specific representations.

## Introduction

**Figure 1.**
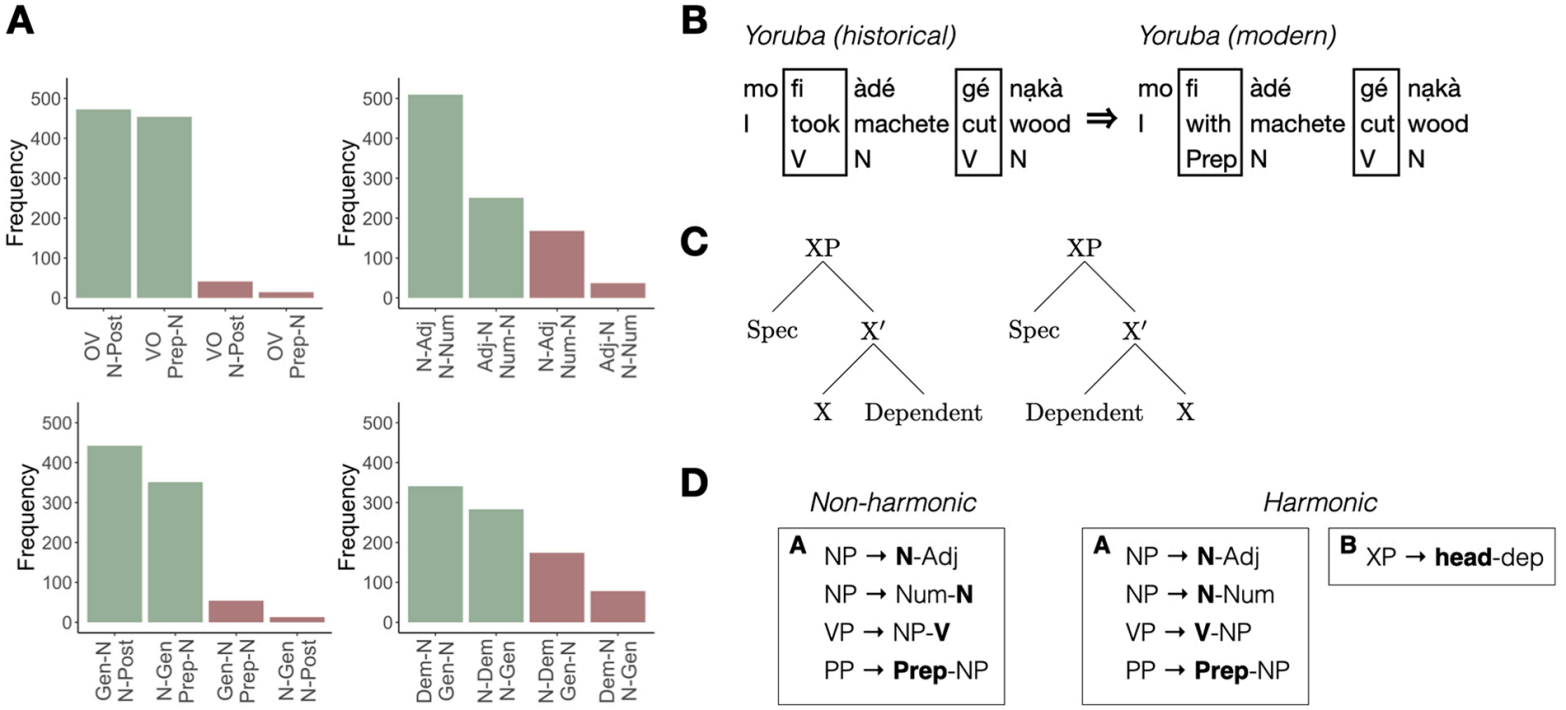
(A) Frequency data from a sample of languages (Dryer & Haspelmath, 2013) illustrating the tendency for harmony across different types of phrases—top left to bottom right: Verb (head), Object (dependent) with Adposition (head), Noun (dependent); Noun (head) and Adjective (dependent) with Noun (head) and Numeral (dependent); Noun (head) and Genitive (dependent) with Adposition (head), Noun (dependent); Noun (head) and Genitive (dependent) with Noun (head) and Demonstrative (dependent). (B) Example data from Yoruba (Givón, 1975) showing grammaticalization pathway of the word *fi* changing from a verb (meaning “take”) to a preposition (meaning “with”). Order of the element relative to its noun dependent stays the same. (C) Standard X-bar representation of head-initial and head-final hierarchical structure illustrating harmony as a linguistic notion; all phrase types in a language could in principle slot into one of these two, with a high-level Head Parameter indicating which is the default structure for a given language (Baker, 2001). (D) Simplified illustration of the number of rules needed to represent a harmonic vs. non-harmonic pattern in language (using just two phrase types). For the non-harmonic grammar, rules for generating both phrase types must be listed separately. For the harmonic grammar, there is an alternative option: a single rule reused across phrases (Culbertson & Kirby, 2016).

The study of human cognition has as its fundamental goal to understand the mechanisms and representations we use to perceive, organise, and communicate about the world around us. The extent to which mechanisms and representations vary across individual humans and human societies has important implications for theories of cognition. Language is an important test bed for these theories since both variation and apparent constraints on variation are extensively documented. For example, languages differ in how they order words in different phrases, but languages with verb-object order (e.g. “kick ball”) tend to have prepositions (e.g., “to school”) and languages with object-verb order (e.g., “ball kick”) tend to have postpositions (e.g., “school to”). In both cases, verb and adposition order is aligned, coming either first or last in the phrase. Such patterns of alignment are found across many types of phrases, a phenomenon known as word order harmony (Figure 1A). There is a vast literature on harmony in linguistics, in part because over time, additional language data has continued to substantiate it, making it one of the most robust cross-linguistic tendencies ever documented. The interest in harmony also reflects the fact that it is a higher-level generalisation that links seemingly distinct grammatical categories, like verbs and adpositions, in a way that potentially highlights the abstract nature of syntax. However, a number of different explanations have, in fact, been proposed for word order harmony, and these reflect very different stories about why human languages look the way they do and have different implications for the nature of human cognition.

One type of explanation argues that harmony is the result of orthogonal, and potentially accidental, patterns of language change and language history. For example, there are common pathways of grammatical change from one type of word to another, like verbs changing to become adpositions (Aristar, 1991; Bybee, 1988; Givón, 1979; Whitman, 2008). This change in word type does not necessarily affect word order. Therefore, if the historical source for adpositions in a language is verbs, then verb and adposition order will by default align (Figure 1B). This kind of alignment does not involve any special cognitive status for harmony—indeed it has been proposed as an alternative explanation since it reflects potentially orthogonal factors (e.g., similarities in meaning) that make certain classes of words good historical sources for other classes. More generally, this kind of work underscores the fact that at least some apparent constraints on language may reflect population-level patterns of change (e.g., mistakes in articulation or perception that do not reflect cognitive representations or processes [Blevins, 2004; Ohala, 1993]) or even noise in the small nonindependent sample of languages to which we now have access (Dunn, Greenhill, Levinson, & Gray, 2011; Gell-Mann & Ruhlen, 2011; Ladd, Roberts, & Dediu, 2014; Piantadosi & Gibson, 2014); they are *not* necessarily a result of individual-level cognitive mechanisms or representations that pick out harmony as preferred over disharmony (Aristar, 1991; Cristofaro, 2017; Whitman, 2008).

Nevertheless, a number of theories have been developed that *do* link harmony to human cognition. In some cases, aspects of cognition implicated are argued to be specific to language (Baker, 2001; Dryer, 1992a; Hawkins, 1994; Vennemann, 1976). For example, one well-known theory is that harmonic order holds between syntactic heads of different categories (Baker, 2001; Chomsky, 1988; Travis, 1984). Verbs are heads of verb phrases (and take nouns and other types of phrases as their syntactic dependents), adpositions are heads of adposition phrases (and take nouns as their dependents), and so on. According to this classic view, heads sit it a particular position in the syntactic structure (i.e., as specified by x-bar theory), and thus they can be governed by a single high-level rule. Language learners use a specialized language acquisition device to set a binary parameter for their language: heads-first or heads-last (Figure 1C). Related theories have argued for a different notion of similarity between the elements that align, or different language-specific mechanisms for deriving harmony, but share the basic idea that consistent alignment is better (Cinque et al., 2017; Dryer, 1992a, 2009; Greenberg, 1963; Hawkins, 1994; Vennemann, 1976). If harmony in language reflects a preference for specific ways of representing and sequencing syntactic categories, then this could be evidence of a specialised linguistic mechanism underlying harmony. Note that this is quite a different kind of explanation to the previous one, which has harmony arising as a by-product of historical processes.

However, a third view has proposed that harmony may have its origins in *simplicity*, a domain-general learning bias. Across many cognitive domains, there is evidence that human learners prefer simpler explanations or hypotheses (Chater & Vitányi, 2003), for instance, those which are more compressible or can be expressed with fewer, shorter rules. Harmonic patterns can be expressed as *a single rule*, either ordering heads relative to dependents or ordering elements that share a similarity along some other dimension (for example, whether the category tends to be “branching”, i.e., be made up of a single item, or a more complex phrase as argued by Dryer, 2009). By contrast, non-harmonic patterns will require additional rules. The particular similarity relation among the words that tend to align is here not at issue, the crucial thing that makes harmony cognitively special according to this view is that it aligns similar categories in a way that makes the system more compressible (Figure 1D). Harmony would then be an instance of a domain-general bias for compressibility interacting with language in a domain-specific way (Culbertson & Kirby, 2016). Language learners acquire grammatical categories like nouns, verbs, and adpositions on the basis of linguistic evidence, and generalizations about order are formed over these language-specific categories. A domain-general simplicity bias works behind the scenes to drive learners to prefer ordering rules that involve consistent alignment of categories that are similar on some relevant dimension—for example, that they are all heads. Over time, the pressure for harmony at each generation will influence how languages change: harmony is more likely to be stably maintained, while disharmonic orders will be more likely to change to harmonic ones (see Kirby, 1999; Thompson, Kirby, & Smith, 2016, for more detailed discussion of weak/defeasible cognitive biases and how they influence typology). Crucially, if this is the right explanation for harmony, then consistent alignment should be preferred not just for linguistic categories but for categories in any other domain or modality. If this is the case, then what we see in language is the realisation, in that particular domain, of a more general cognitive bias. In other words, the patterns of word order we see in the world’s languages are both explained by and reveal a general organising principle of cognition.

Here, we follow a long tradition of exploring the cognitive mechanisms of language learning with artificial grammar, or pattern learning experiments. In recent years, such experiments have been used to investigate the link between cognition and the frequency of patterns in the world’s languages (e.g., Culbertson & Adger, 2014; Culbertson, Franck, Braquet, Barrera Navarro, & Arnon, 2020; Fedzechkina, Jaeger, & Newport, 2012; Maldonado & Culbertson, 2020; Martin & White, 2019). For example, work using artificial language learning experiments suggests that harmony between certain types of phrases is indeed preferred by learners. Both adult and child learners reproduce harmonic orders of nominal modifiers but change non-harmonic patterns (e.g., where adjectives follow nouns and numerals precede them) into harmonic ones (Culbertson & Newport, 2015, 2017; Culbertson, Smolensky, & Legendre, 2012). Importantly, this result holds even for speakers whose native language is non-harmonic in just this way (Culbertson et al., 2020). Artificial language learning experiments have also shown that learners generalise the order of verb and object they are trained on to the order of adposition and noun (Wang, Kirby, & Culbertson, 2021). If they learn that verbs come first in a verb phrase, they will assume prepositions; if they learn that verbs come last, they will assume postpositions, irrespective of their native language. These studies therefore already suggest that harmonic patterns are cognitively preferred in linguistic stimuli that resemble the kinds of natural language phrases where harmonic alignment tends to hold.

Whether evidence for harmonic patterns reflects a domain-general bias for compressibility is less clear. A large body of literature has examined the domain-general nature of learning mechanisms using artificial grammar experiments. These studies have reported both similarities and differences across stimulus modalities. For example, the ability to distinguish grammatical from ungrammatical sequences generated by finite state grammars, or to track transitional probabilities in continuously presented stimuli, has been demonstrated across visual (e.g., Fiser & Aslin, 2002; Kirkham, Slemmer, & Johnson, 2002), auditory (e.g., Milne, Petkov, & Wilson, 2018), and tactile (e.g., Conway & Christiansen, 2005) domains. In addition, a number of studies show that learning is generally better when the stimulus modality matches its typical mode of presentation: sequentially presented for auditory stimuli and simultaneously presented for visual stimuli (e.g., Conway & Christiansen, 2009; Emberson, Conway, & Christiansen, 2011; Lukics & Lukács, 2022; Mahar, Mackenzie, & McNicol, 1994; Saffran, 2002). Finally, there is some evidence that implicit learning of grammar-like rules is better when the stimuli are linguistic rather than non-linguistic, at least in the auditory domain (Lukics & Lukács, 2022). However, most these studies focus on general ability across domains and (cf. Saffran, 2002), while here, we are interested in whether differences in learnability based on particular stimulus patterns generalise across domains. That said, previous research on feature-based chunking and memory provides some potential support for our hypothesis about the domain-general relationship between category learning, order, and compressibility. First, category learning experiments show that more compressible categories of visual and auditory stimuli are easier to learn (e.g., Carr, Smith, Culbertson, & Kirby, 2020; Feldman, 2000; Moreton, Pater, & Pertsova, 2015). There is also evidence that more compressible lexicons are easier to learn (Kirby, Tamariz, Cornish, & Smith, 2015). Further, previous studies have shown that list memorisation is facilitated by shared features among adjacent shapes (e.g., Brady, Konkle, & Alvarez, 2009; Chekaf, Cowan, & Mathy, 2016). While none of these studies directly targets ordering regularities that resemble harmony, they nevertheless suggest that rule learning is tied to simplicity.

**Figure 2.**
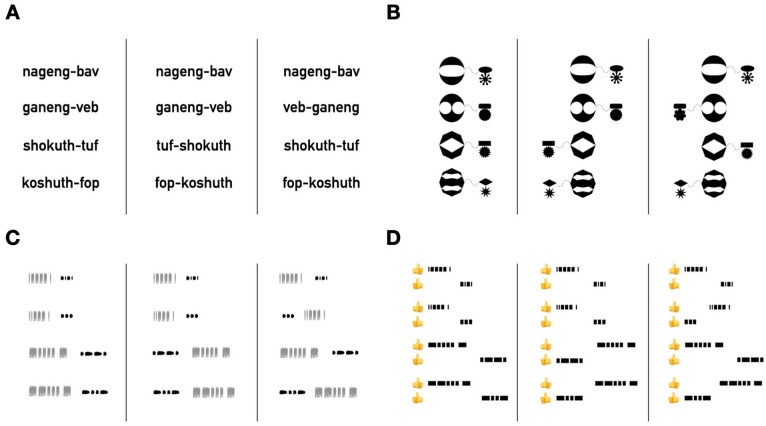
Example pattern sequences for all four stimulus domains. Four patterns out of a total of 64 sequences for an example set of sequences in each condition are shown. Left-hand group: example stimuli in the *harmonic* condition. Middle group: example stimuli in the *non-harmonic across heads* condition. Right-hand group: example stimuli in the *non-harmonic within heads* condition. (A) Non-word sequences. Heads are two-syllable words, and dependents are single syllables. (B) Shape sequences. Heads are large shapes, and dependents are small shapes. (C) Auditory sequences. Gray fuzzy marks indicate broad-band noise; dark solid marks indicate tones. The length of a mark indicates whether the sound is long or short. Heads are longer sequences than dependents. Here also the use of noise versus tones helps delineate between heads and dependents. (D) Tactile sequences. Each mark indicates a sensation being transmitted to either the participant’s left or right thumb. The length of the mark indicates whether the sensation is long or short. As in the auditory stimuli, heads are longer sequences. The distinction between heads and dependents is also signalled by which thumb is being stimulated.

To summarise, previous literature suggests that harmony is preferred in linguistic artificial language learning tasks. In addition to this, research on learning across modalities provides intriguing evidence for the domain-general role of compressibility (see also Chater &Vitányi, 2003), while at the same time suggesting that learning may also be subject to modality-specific effects (see also Christiansen, 2019; Frost, Armstrong, & Christiansen, 2019). In our experiments, we test the domain-general nature of harmony with these previous studies in mind. We sample from a range of stimulus types, including visual, auditory, and tactile. We start with stimuli that are closer to those used in previous studies on linguistic harmony (visually presented nonsense words) and move to stimuli that are far less similar to language (tactile pulses). In each stimulus domain we created harmonic and non-harmonic ordering patterns. In natural language, harmonic ordering patterns involve learned syntactic categories like verbs and adpositions. Our stimuli involved ordering novel categories of stimuli with easy-to-perceive similarities, and participants learned these categories at the same time as their order. In all stimulus domains, we created four sets of elements. Two types we refer to as “heads,” each paired with its own matching “dependents” (Figure 2). Heads were distinguished from dependents based on salient, domain-specific dimensions—for example, size or length. At the same time, head-dependent sets were distinguished based on orthogonal dimensions–for example, shape or speed. These domain-specific distinguishing characteristics do not, of course, make our non-linguistic stimuli “heads” and “dependents”—as described above, we take these to be language-specific categories. However, they are intended to function in a similar way to kinds of characteristics that distinguish grammatical categories from one another in natural languages: grammatical categories have features (e.g., form, meaning, distribution) that distinguish them from other categories; heads can typically appear on their own without dependents, are often claimed to “select” particular dependents, and often share formal features with them (e.g., they exhibit morphological agreement, Zwicky, 1985). In a set of follow-up experiments, we return to the specific characteristics that distinguish categories in our stimuli and how these relate to the notions of similarity that drive linguistic harmony. To preview, we do not expect the specific category features to impact the preference for harmony—since we hypothesise that the preference is based on a domain-general notion of compressibility. All that is required is some property or properties that distinguish one category from the other. Indeed, that is what we find.

In the *harmonic* conditions of our studies, both head-dependent sets had the same order—that is, heads first or heads last. In the *non-harmonic across heads* conditions, the head elements in one set occurred first but last in the other. Finally, in the *non-harmonic within heads* conditions, the heads within one set could occur both first or last relative to the dependents. If harmony is driven by a domain-general simplicity bias, then across all stimulus domains, the harmonic conditions should be easiest, followed by the non-harmonic across heads conditions, with the non-harmonic within heads conditions being hardest to learn. This is because the harmonic conditions require learning only a simple ordering principle (head-first or head-last); the non-harmonic across heads conditions require learning a more complex pattern, differing by head category; and the non-harmonic within heads conditions require learning the most complex set of patterns, differing by each category of dependent per head. In some ways, the prediction that harmonic order should be simpler to learn than non-harmonic order may seem trivial. However, the deeper point that highly abstract features of language structure—like the alignment of syntactic heads across very different types of phrases—can be influenced by domain-general mechanisms like simplicity is not at all trivial (see, e.g., Berwick & Chomsky, 2017; Christiansen, Chater, & Culicover, 2016; Evans & Levinson, 2009, on the debate around domain specificity in linguistic explanation).

## Experiments 1–4: Order learning across modalities

In our first set of experiments, we compare harmonic and non-harmonic ordering patterns instantiated in non-word strings, sequences of shapes, auditory sequences, and tactile sequences. Our goal is to assess whether differences in learnability across ordering conditions are the same across all four modalities—ranging from more similar to language (non-words) to less similar to language (tactile sequences). As discussed previously, in order to learn ordering patterns that operate at the level of categories, participants must learn to distinguish the categories themselves. For this reason, we did not design stimulus sets that were maximally similar across domains (cf. Conway & Christiansen, 2005, who do exactly this in the context of a study on implicit learning ability across modalities). Because categories and relations between them are likely easier to learn in some domains compared to others (e.g., Conway &Christiansen, 2005; Mahar et al., 1994), we prioritised creating sufficiently salient distinctions for learners to pick up on. While the specific features of the stimuli used are described in detail below, a few general characteristics remain constant across domains. First, heads were always presented to participants in isolation before they were presented in combination with dependents. Second, heads and dependents differed in set size, with fewer heads than dependents. It also mirrors, for example, the fact that heads in natural language are more likely to be closed class categories (e.g., adpositions) and are less likely to be multi-word (resulting in fewer types). Categories of heads and the dependents that co-occur with them also share formal features (as described in detail below) similar to how heads and dependents might exhibit concord (or agreement) with each other. These characteristics both mimic differences between heads and dependents in language and provide salient cues to the high-level distinction that is relevant for participants in our studies.

### Methods

Experimental stimuli as well as code needed to run experiments and analyze data can be found (https://osf.io/jyuwz/?view_only=4c84c94729e849d0bd1d65343deea2ba). Minor deviations from the preregistration are noted below.

#### Participants

Participants in the non-word, shape, and auditory experiments were native-English speakers who self-identified as monolingual on the Prolific platform. Due to the nature of the apparatus needed for the tactile experiment (see below), participants in this experiment were run in person in a quiet room at the University of Verona by the second author, an expert in tactile sequence learning. These participants were native Italian speakers. This means that the experiments differ in the native language of the participants. In principle, one might be concerned that experience with harmony or non-harmony in the native language might influence behavior in these studies (e.g., as found for a different cross-linguistic tendency, Martin & Culbertson, 2020). However, note that almost all languages feature some harmonic and some non-harmonic patterns. For example, English has harmonic alignment of verbs and their dependents, and adpositions and their dependents, but non-harmonic alignment of verbs and their dependents and nouns and their dependents (verbs precede, nouns follow). Similarly, Italian aligns verbs and adpositions but has a mix of harmonic and non-harmonic ordering of nominal dependents (numerals precede nouns, but adjectives follow). In addition, previous experimental studies of harmony have in fact found a preference for harmony independent of prior linguistic experience (e.g., Culbertson et al., 2020). Further, the cross-domain nature of our stimuli means that in some cases the patterns to be learned are very distant indeed from language. Therefore, if we see the same results across domains, linguistic experience is unlikely to be the explanation. We return to this in the general discussion below. All participants were paid at a rate of 9 GBP/hr (10€). A total of 306 participants were run across all stimulus domains and conditions (non-word sequences: 25 harmonic, 25 non-harmonic across heads, 25 non-harmonic within heads; shape sequences: 25 harmonic, 25 non-harmonic across heads, 26 non-harmonic within heads; auditory sequences: 24 harmonic, 30 non-harmonic across heads, 25 non-harmonic within heads; tactile sequences: 26 harmonic, 25 non-harmonic across heads, 25 non-harmonic within heads).

#### Materials

Materials in all stimulus domains were created by developing two major categories of elements, which we call heads and dependents. In addition to the general characteristic described previously, head elements were distinguished from dependent elements based on specific characteristics that differed by domain: length (non-word, auditory, and tactile stimuli), size (shape stimuli), type of sound (auditory stimuli), or hand (tactile stimuli). Heads and dependents were separated into two classes (called Head1, Head2, and Dep1, Dep2 below). Akin to grammatical categories in language, these classes were designed to be perceptually distinct in each stimulus domain. For example, round shapes vs. angular shapes, or fast tempo vs. slow tempo sounds. Heads and dependents were always matched for class—for example, round heads went with round dependents, and angular heads went with angular dependents. As mentioned previously, these distinctions were designed to be easy to perceive since learning the relevant categories is key to learning the ordering patterns, but category learning was *not* the main question of interest here.

Each class of dependents was further divided into two subclasses (e.g., Dep1a, Dep1b below); however, these were designed to be more similar to individual lexical items within a given grammatical category language. Therefore, these subclasses were less obviously distinct from one another.

In all stimulus sets, there were 8 heads (4 of each type) and 16 dependents (8 of each type, 4 of each subtype). This meant a total of 64 possible combinations. The sequential order for a given combination was dependent on the condition (see Manipulation).

##### Non-word stimuli

Stimuli were comprised of letter strings. Heads were longer (2 syllables) than dependents (1 syllable). Heads within a given category used a consistent set of vowels (Head1: a,e; Head2: o, u) and consonants (Head1: voiced sounds n, ng, g; Head2: voiceless sounds sh, k, th) in the string. Similarly, dependents paired with each head category used the same vowels (Dep1: a, e; Dep2: o, u) and consonant types (Dep1: voiced sounds b, v, d, z; Dep2: voiceless sounds p, f, t, s). Subtypes of dependents used different vowels (Dep1a: a, Dep1b: e; Dep2a: u; Dep2b: o). The full set of heads and dependents is shown in Table 1.

**Table 1.** Non-word head and dependent stimuli.

Heads		Dependents	
Head1	nageng, negang,	Dep1a	bav, baz, dav, daz
	genang, ganeng	Dep1b	veb, ved, zeb, zed
Head2	shukoth, shokuth,	Dep2a	puf, pus, tuf, tus
	koshuth, kushoth	Dep2b	fop, fot, sop, sot

##### Shape stimuli

Stimuli were comprised of shapes. Heads were larger than dependents. Heads within a given category were either round shapes with round insets, or angular shapes with angular insets. Similarly, dependents paired with each head category were comprised of a set of two smaller round shapes or two smaller angular shapes. Subtypes of dependents differed in the exact two shapes used (Dep1a: oval with one of four skinny flower shapes beneath, Dep1b: rounded rectangle with one of four fat flower shapes beneath; Dep2a: diamond with one of four skinny star shapes beneath; Dep2b: a rectangle with one of four fat star shapes beneath). The full set of heads and dependents is shown in Table 2.

**Table 2.** Shape head and dependent stimuli.

Heads		Dependents	
Head1	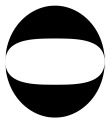 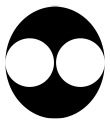 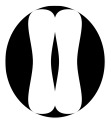 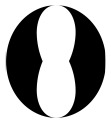	Dep1a	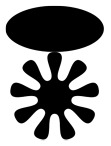 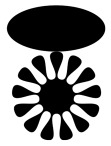 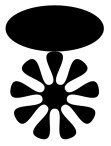 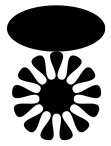
		Dep1b	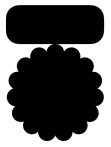 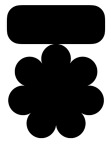 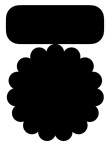 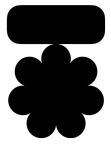
Head2	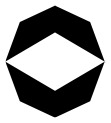 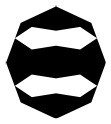 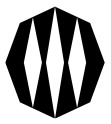 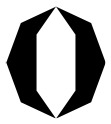	Dep2a	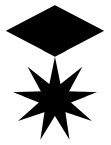 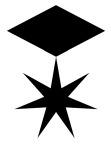 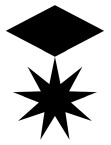 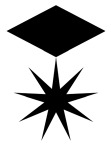
		Dep2b	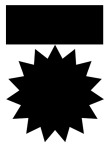 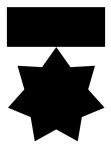 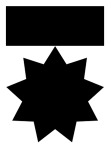 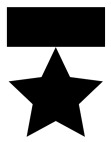

##### Auditory stimuli

Auditory stimuli were designed to be non-language-like and were comprised of sequences of morse code-like dots and dashes. Heads were comprised of longer sequences of dots and dashes than dependents. In order to disguise the morse-like nature of the stimuli (in case participants perceived the sequences as a language) and make the two high-level categories easier for participants to distinguish, heads and dependents were also distinguished based on the type of sound they were made up of, either pure tones or band-pass filtered noise (counterbalanced across participants), and dots were produced at 800 hz and dashes were produced at 400 hz. Heads within a given category were made up of either fast tempo sounds (Head1: 40 ms per dot, 120 ms per dash, 40 ms between sounds, 120 ms between morse letters, and 240 ms between heads and dependents) or slow tempo sounds (Head2: double the timings for all). Head categories were additionally differentiated based on whether they started with a dot or a dash. Dependents paired with each head category matched them in the tempo of each sound (Dep1: fast tempo, Dep2: slow tempo). Subtypes of dependents either started with a dot or a dash (either matching or not the head). The dot/dash patterns used for all heads and dependents is shown in Table 3, along with corresponding morse-code letter sequences for each element for illustrative purposes.

**Table 3.** Auditory/tactile head and dependent dot and dash patterns. Auditory experiment: dots and dashes mapped to either pure tones for heads and band-pass filtered noise for dependents, or vice versa. Tactile experiment: head sequences were felt on the right thumb and dependent sequences on the left, or vice versa.

Heads		Dependents			
Head1		Dep1a		Dep1b	
⋅___⋅	(1e)	_⋅_⋅	(c)	⋅_⋅	(r)
⋅⋅__⋅	(2e)	__⋅_	(q)	⋅⋅⋅	(s)
⋅⋅⋅__⋅	(3e)	_⋅⋅	(d)	⋅⋅_⋅	(f)
⋅⋅⋅⋅_⋅	(4e)	__⋅	(g)	⋅_⋅⋅	(l)
Head2		Dep2a		Dep2b	
_⋅⋅⋅⋅_	(6t)	_⋅⋅_	(x)	⋅__⋅	(p)
__⋅⋅⋅_	(7t)	_⋅__	(y)	⋅⋅⋅_	(v)
___⋅⋅_	(8t)	_⋅_	(k)	⋅⋅_	(u)
____⋅_	(9t)	____	(o)	⋅__	(w)

##### Tactile stimuli

Stimuli were comprised of sequences of morse code–like dots and dashes transmitted to participants’ thumbs as 120 Hz vibratory pulses via wireless Bluetooth bone conduction headphones (AfterShokz Aeropex). Headphone transducers were secured to participants’ left and right fingertips through nitrile thimbles. Heads were comprised of longer sequences of dots and dashes than dependents. To help participants distinguish these high-level categories, heads and dependents also differed based on whether they were felt on the right or left thumb (counterbalanced across participants). Heads within a given category were made up of either fast tempo vibrations (Head1: 40ms per dot, 120 ms per dash, 40 ms between sounds, 120 ms between morse letters, and 240 ms between heads and dependents) or slow tempo vibrations (Head2: double the timings for all). Head categories were additionally differentiated based on whether they started with a dot or a dash. Dependents paired with each head category matched them in the tempo of each sound (Dep1: fast tempo, Dep2: slow tempo). Subtypes of dependents either started with a dot or a dash (either matching or not the head). The dot/dash patterns used for all heads and dependents is the same as for the auditory stimuli (see Table 3).

##### Manipulation

Participants were randomly assigned to one of three conditions: harmonic, non-harmonic across heads, and non-harmonic within heads. In the harmonic condition, the relative order of heads and dependents was consistent across all sequences in the language. Participants in this condition were randomly assigned to heads first or heads last. The harmonic condition would be parallel to a language in which verbs and adpositions were aligned—i.e., places in the same order relative to their dependents (e.g., English, Turkish). In the non-harmonic across-heads condition, the order of heads and dependents differed across the two categories of heads. For example, participants might see Head1-Dep1 for all category 1 pairs but Dep2-Head2 for all category 2 pairs. Which category had heads first versus last was randomly assigned for each participant in this condition. This condition would be parallel to a language with a non-harmonic order of verbs and adpositions—for example, verbs before their dependents but postpositions after (e.g., Farsi, Tigringa). In the non-harmonic within heads condition, for each category of head, one subtype of dependents came first and the other last. For example, Head1-Dep1a but Dep1b-Head1, and Dep2a-Head2 but Head2-Dep2b. The particular dependent subtype that occurred first versus last for each head category was randomly assigned for each participant in this condition. This condition would be parallel to a language in which particular verbs were ordered first relative to some dependents but last for other, and similarly for adpositions (similar to Mandarin, which has both prepositions and postpositions used to convey different meanings). Example stimuli for each condition in each stimulus domain are shown in Figure 2. Example complete stimulus lists for each condition in the non-word sequence experiment can be found in the Supplementary Materials.

##### Procedure

The experiment was presented in a web browser using jspysch (de Leeuw, Gilbert, and Luchterhandt, 2023). Participants were informed that they would be learning to recognize two new types of sequences (“letter sequences,” “shape sequences,” “sound sequences,” or “tactile sequences that you will feel through your thumbs as a series of pulses”). They were told that each type of sequence has a “shorter sequence” (“smaller sequence” for the shape condition) associated with it, and their task was to learn to recognize how the short (small) sequences attach to the long (large) ones.

They were then given examples of the two types of heads in isolation. These were either presented visually on the screen (non-word and shape sequences) or could be played one at a time so that participants could hear (auditory sequences) or feel (tactile sequences) them. After this, they were trained on head and dependent sequences one at a time. On each trial, a head and dependent sequence was (dis)played, and after 1500 ms, a button labelled with “Next” appeared and participants clicked to advance to the next trial. Training consisted of 64 trials (one repetition of each possible combination of head with dependent in the language). The order of presentation was randomized across trials for each participant.

After this training, participants were tested on the ordering pattern in the language. In each order testing trial, two sequences were (dis)played. In the non-word and shape experiments, these were displayed visually next to each other on the screen. In the auditory experiment, the sounds were played one at a time, with the two sounds being represented on the screen as speaker icons, shown next to each other on the screen. When the first sound was playing, the first speaker was black, and the second speaker was greyed out. When the second sound was playing, the second speaker was black, and the first was greyed out. In the tactile experiment, the same procedure was used, but rather than two speakers, two abstract waveform images were displayed, one above the other. Participants were instructed to choose which was a possible configuration of the sequences they had learned about. In the non-word, shape, and auditory experiments, they simply clicked the corresponding image (letter sequence, shape sequence, or speaker). In the tactile experiment, participants were instructed to choose the top image by typing “g” or the bottom image by typing “b.” This was because participants could not comfortably use a mouse while the bone-conduction headphones were secured to their thumbs. Given a neutral thumb position in front of a keyboard, “g” and “b” are easy to reach, and because they are aligned one on top of the other, they do not interfere with the left-right distinction used in the stimuli. Testing consisted of 64 trials, one repetition of each correct combination of head with dependent in the language, paired with a sequence that reversed the order. For example, if the participant was in a harmonic condition, and the correct sequence was of the form Head1-Dep1a, then the incorrect sequence was of the form Dep1a-Head1, with the two parts of the correct sequence swapped. The order of presentation, as well as whether the correct sequence was the first/top or second/bottom, was randomized across trials for each participant.

Finally, participants were tested on their knowledge of the dependencies in the language. The procedure and instructions were exactly the same as for the ordering test. This test consisted of 32 trials, chosen randomly from a set of 64, consisting of one repetition of each correct combination of head with dependent in the language, paired with a sequence in the same order but with the wrong type of dependent. For example, if the correct sequence was of the form Head1-Dep1a, then the incorrect sequence was of the form Head1-Dep2a, where the head element was the same, but the dependent element was the corresponding element from the Dep2a set. In other words, the incorrect dependent was always chosen from the same subtype (a or b) of the other dependent category. The order of presentation, as well as whether the correct sequence was the first/top or second/bottom, was randomized across trials for each participant.

## Results

**Figure 3.**
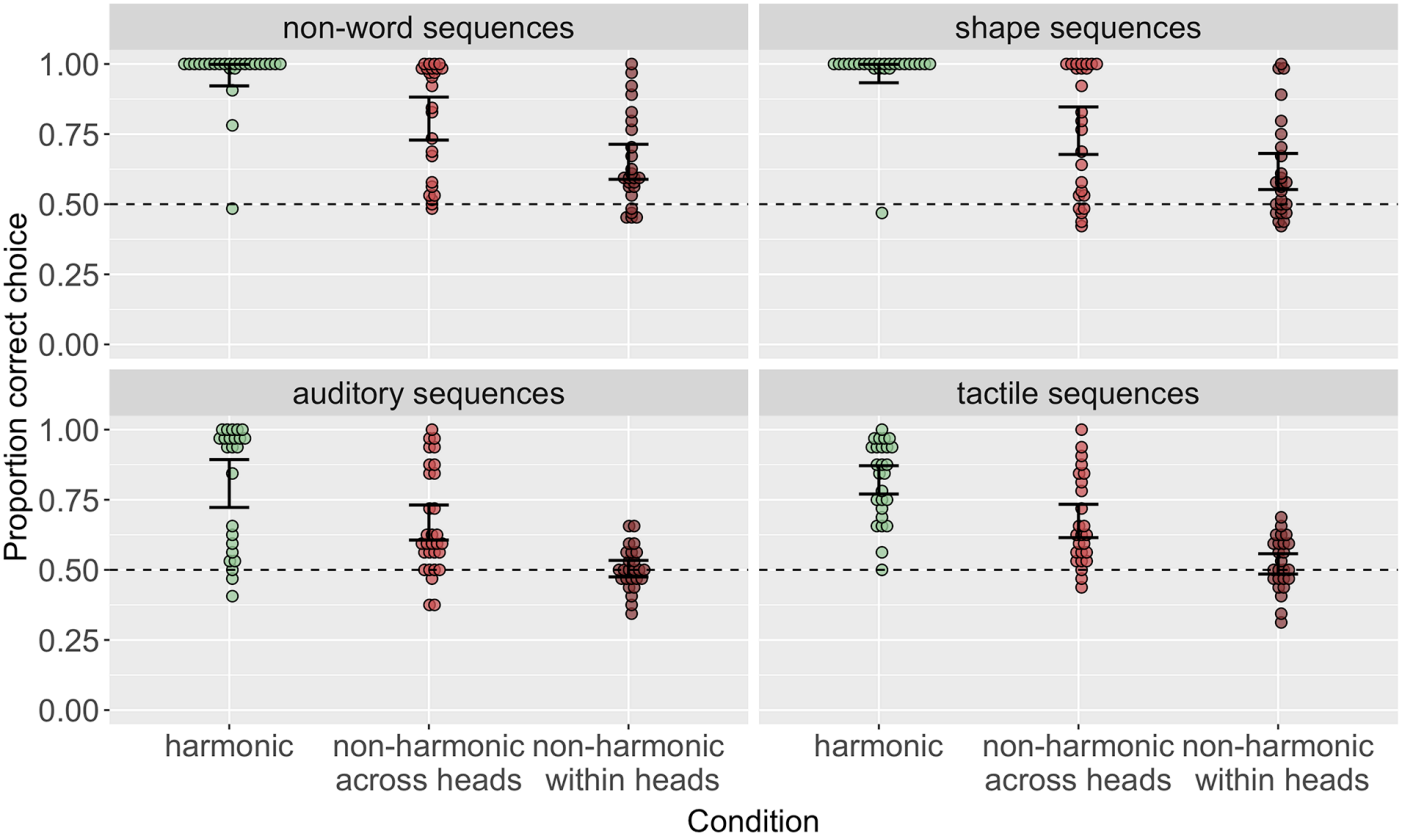
Order accuracy across conditions in each stimulus domain. Across all stimulus domains, harmonic conditions are learned with highest accuracy; non-harmonic within heads conditions are learned with lowest accuracy.

Figure 3 suggests that, as predicted, learning accuracy was highest in the harmonic conditions and lowest in the non-harmonic within heads conditions across all stimulus domains. Following our pre-registered analysis plan here, we assessed this statistically using logistic mixed-effects models, run in R R Core Team (2022) using lme4 (Bates, Mächler, Bolker, and Walker, 2015). For each experiment, we ran three models: one intercept-only model, one model predicting accuracy by condition with the harmonic condition as the baseline, and one model comparing only the two non-harmonic conditions, with non-harmonic across heads as the baseline. We used a likelihood ratio test comparing the first two models in each case to assess the main effect of the condition. We report estimates, standard error, and *p*-values from the second two models. These models confirmed the predicted pattern of results in all cases, with participants in the harmonic conditions achieving significantly higher accuracy than participants in both non-harmonic conditions and participants in the non-harmonic within heads having the lowest accuracy (see SI for full model results).

We also ran preregistered follow-up exploratory analyses investigating whether the differences between conditions were of a similar magnitude across stimulus domains. Figure 3 suggests that the pattern of results was very similar in the non-word and shape sequences conditions and in the auditory and tactile conditions but might differ to some degree between these types. Indeed, although the best and worst learned ordering conditions remain the same across all experiments, a model predicting order accuracy by condition (Helmert coded, with harmonic as the baseline), visual vs. non-visual stimuli (treatment coded with non-visual as the baseline), and their interaction revealed a significant main effect of condition $(\chi^{2}(1)=135.83,p<0.001$ ; harmonic vs. non-harmonic: β=−0.54±0.14,p<0.001 ; non-harmonic across vs. within: f β=−0.46±0.08,p<0.001) , confirming our pre-registered individual analyses for each experiment reported in the SI. The model also revealed a significant main effect of visual vs. non-visual stimuli $\left(\chi^{2}(1)=74.71,\beta =1.78\pm 0.18,p<0.001\right)$ and a significant interaction (condition x visual vs. non-visual interaction: $\chi^{2}\left(2\right)=39.44,p<0.001)$ . The interaction was likely driven by the very high performance in the harmonic conditions in the two visual experiments (effect of harmonic vs. non-harmonic across heads in visual, as compared to non-visual domains: β=−1.13±0.23,p<0.001 ; effect of harmonic+non-harmonic across heads vs. non-harmonic within heads in visual, as compared to non-visual domains: β=−0.54±0.12,p<0.001 ). This suggests that while harmonic patterns are easier to learn than non-harmonic patterns across all the stimulus domains we tested, auditory and tactile stimuli made for a more difficult learning task than the non-word and shape stimuli.

**Figure 4.**
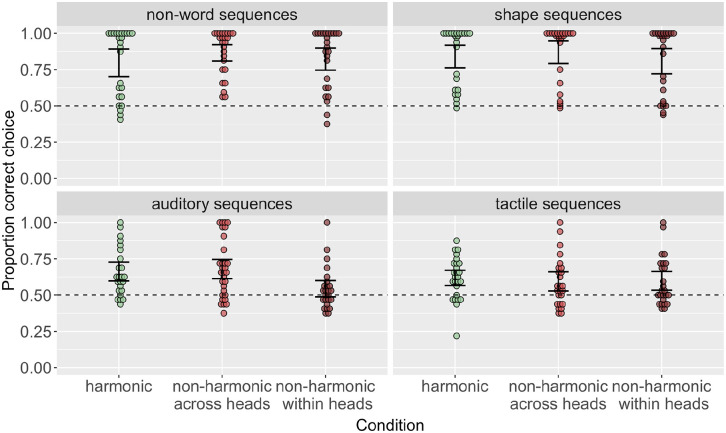
Dependency accuracy across conditions in each stimulus domain. Dependencies are generally learned equally well across conditions, though learning is better for non-word and shapes sequences compared to auditory and tactile sequences).

In order to test whether participants were picking up on the general structure of the stimuli sets and not merely attending to the ordering patterns, we also explored whether participants learned the dependencies—that is, which head elements could occur with which dependent elements—across all conditions in each stimulus domain (Figure 4). Here, the dependencies to be learned are constant across conditions in a given stimulus domain. As with our analysis of order accuracy, we ran model comparisons for all experiments to test whether accuracy differed by condition. In three out of four stimulus domains (non-words, shapes, tactile sequences), as expected, learning was above chance and did not differ across conditions. In the auditory sequence experiment, there was an effect of condition $\left(\chi^{2}(2)=8.11,p=0.02\right)$ , driven by worse performance in the non-harmonic within heads condition (see SI for full results of all models). These results suggest that, in general, the dependencies were learned across conditions in all stimulus domains, and therefore, participants are learning features of the sequences beyond merely order. Furthermore, this suggests that the robust differences we see between conditions in all domains are not likely to be driven by accidental differences in dependency learning. However, as with order, dependency learning was more difficult in the auditory and tactile domains. A model predicting dependency-learning accuracy by condition (Helmert coded, with harmonic as the baseline), visual vs. non-visual stimuli (treatment coded with non-visual as the baseline), and their interaction revealed a significant main effect of visual vs. non-visual $\left(\chi^{2}\left(1\right)=108.65,\beta =2.12\pm 0.20,p<0.001\right)$ stimuli. This result suggests that, as with order, dependency learning was more difficult for the auditory and tactile stimuli as compared to the non-word and shape stimuli.

### Discussion

In the experiments reported previously, we tested the domain-general nature of the bias for word order harmony and the cross-linguistic tendency for consistent alignment between elements like verbs and adpositions across phrases. In linguistics, there is a long history of competing explanations for harmony, which range from accidents of history (e.g., Dunn et al., 2011) to processes of language change that are independent of order (e.g., Aristar, 1991; Whitman, 2008) to domain-specific constraints on syntactic representations (e.g., Baker, 2001; Travis, 1984). The hypothesis that harmony reflects a domain-general bias for simplicity or compressibility has been proposed more recently (Culbertson & Kirby, 2016) but is in line with older ideas about the link between rule consistency and harmony (e.g., Dryer, 2009; Greenberg, 1963; Vennemann, 1976). To test this hypothesis experimentally for the first time, we created artificial grammars in four distinct domains and modalities, each with two high-level categories of similar elements—which we labelled “heads” and “dependents”—whose order was harmonic or not. In harmonic conditions, different types of heads occurred in a consistent order relative to their dependents. In non-harmonic conditions, they did not. We found that in all cases, harmonic orders were easier to learn, and non-harmonic orders were harder. We also found a consistent difference between two types of non-harmonic patterns, one in which order was non-harmonic across different types of head elements and another where order was non-harmonic even within heads of a given type.

Notably, there were differences in overall learning of both order and dependencies (i.e., which heads co-occurred with which dependents) across modalities. In particular non-word sequences and shape sequences were learned better than auditory or tactile sequences. These differences follow those found in previous work on artificial pattern learning to some degree. For example, previous work has found that tactile sequences are harder to learn than matched types of sequences in other modalities (e.g., Conway & Christiansen, 2005; Mahar et al., 1994). However, previous work has often found that sequentially-presented auditory sequences are either similarly learnable or more learnable than visual sequences (Conway & Christiansen, 2005, 2009; Lukics & Lukács, 2022; Saffran, 2002). It is worth pointing out, though, that learnability differences are generally found when the visual stimuli are presented sequentially, not simultaneously—as in our shapes and non-words experiments—(e.g., Conway & Christiansen, 2005; Saffran, 2002), or when the auditory stimuli are linguistic—which ours are not (Lukics & Lukács, 2022). These findings suggest that true differences in ability to learn across modalities are largely driven by core mechanistic differences in how we perceive and process stimuli across domains: visual stimuli are learned better when they are presented simultaneously, auditory stimuli when they are presented sequentially, mirroring how we typically process these. Our stimuli followed these domain-typical presentation styles. It, therefore, seems likely that differences in learning across modalities in our task were driven by differences in the salience or similarity of the categories we created.

Perhaps unsurprisingly, previous work in pattern learning has shown that salient perceptual grouping cues help participants learn dependencies in non-linguistic auditory stimuli (e.g., Creel, Newport, & Aslin, 2004). Such grouping cues are also ubiquitously present in language (e.g., in prosody, agreement, and features like predictice dependencies; Morgan, Meier, & Newport, 1987; Saffran, 2002). Recall that in our experiments, we did not aim to exactly match the stimulus categories across domains; rather, we sought to ensure high salience in all domains. Nevertheless, we may not have succeeded in doing so to the same degree across modalities. For example, it may have been that the distinction between heads and dependents was less salient in the auditory and tactile stimuli. This would have made heads more difficult to distinguish from dependents (making their relative order more difficult to learn). The features shared between heads and dependents of a given type could also have been less salient in the auditory and tactile stimuli. This would have made both order and dependency relations more difficult to learn.^1^

All that being said, importantly, the apparent differences in learnability across modalities did *not* lead to differences in the effect of interest here. Regardless of whether learners are acquiring order in visual, auditory, or tactile stimuli, and regardless of whether the stimuli are more (e.g., words) or less (e.g., tactile pulses) linguistic, harmony was preferred. Below we ask to what degree the specific features we used to determine what was a head versus a dependent might have impacted our findings. This is an important question since we are trying to link our findings to an apparently quite abstract theoretical concept in syntax. Therefore, in principle, it could be that the nature of the categories is critical to the preference for harmony. However, our hypothesised explanation, a domain-general preference for simplicity, and our findings so far, that stimulus domain or modality does not impact the preference for harmonic order, suggest this may not be the case.

## Experiments 5–6: What’s in a head

In the experiments presented above, we focused on creating sets of stimuli in which there were two salient categories that could align with each other. We called these categories heads and dependents, but this was, in fact, somewhat arbitrary (setting aside the fact that the set of heads was smaller and that we presented them individually at the start of the experiment). For example, we called the elements that were longer/bigger heads and the ones that were shorter/smaller dependents. But we could have equally reversed that. For the three ordering conditions we compared, it would not have made any difference. In our harmonic conditions, similar elements always align: one class of similar elements (e.g., the bigger ones) is on one side, and the other class (e.g., the smaller ones) is on the other side. This can be stated either in terms of heads (e.g., heads first) or dependents (e.g., dependents last).

Characterisations of harmony in natural language typically share this property. Regardless of the theory, harmony involves consistent alignment of two classes of categories across different types of phases. In theories of harmony that are based on a head-dependent asymmetry (e.g., Baker, 2001; Chomsky, 1970; Travis, 1984), heads align with heads and dependents with dependents. It is always possible to state the ordering pattern with respect to either category. In alternative theories of harmony, the categories that align are specified according to different criteria. For example, the branching-direction theory of harmony (Dryer, 2009) argues that lexical categories (which are comprised of a single word) align with other lexical categories; phrasal categories (which can be comprised of multiple words) align with phrasal categories. Again, ordering can be stated in terms of either. In dependency grammar (e.g., Nivre, 2005), where words enter into a symmetrical dependency relation with each other, harmony would simply be diagnosed as consistent alignment of some set of dependents with some other set.

The differences between these theories are also telling. They indicate a general lack of theoretical consensus on exactly what characterises the categories that exhibit word order harmony. In one case, it is an abstract structural position; in another, it is complexity or length. However, there is also evidence that semantic similarity is relevant in determining alignment. For example, cross-linguistically, semantically similar elements like verbs and adpositions are more likely to exhibit alignment in order than less semantically similar elements like verbs and stative adjectives (e.g., “hit the ball” with “behind the ball” vs. “hit the ball” with “red ball”; Dryer, 1992a). Crucially, evidence from artificial language learning experiments has found that when adjectives are more verb-like, participants are more likely to infer harmonic order between them (e.g., “hit the ball” and “twisted ball”; Wang, Kirby, & Culbertson, 2024). This suggests that less abstract notions of similarity are also important factors in syntactic harmony. Nevertheless, one might wonder whether the features we used to distinguish our two categories are insufficiently parallel to natural language. For example, while we modelled some distinctions on work defining heads in linguistics (e.g., heads appeared in isolation and were fewer in number; Zwicky, 1985), perhaps using size, length, or other perceptual features is simply too different from the bases of similarity that are relevant in syntax. Here, we present an additional set of experiments that test the degree to which the pattern of results we found in the experiments above depends on the particular notion of similarity that determines category membership. In particular, we ask whether we can replicate our results in a set of stimuli that are more symmetric—that is, where “heads” are not longer/bigger than dependents.

### Participants

Participants were native-English speakers who self-identified as monolingual on the Prolific platform. Participants were paid at a rate of 10.20 GBP/hr. A total of 119 participants were run (non-word sequences: 22 harmonic, 20 non-harmonic across heads, 19 non-harmonic within heads; shape sequences: 19 harmonic, 19 non-harmonic across heads, 20 non-harmonic within heads).

### Materials

#### Non-word stimuli

Stimuli were comprised of letter strings. As before, different vowels and consonants provided a cue to the two different categories of heads and dependents. The distinction between heads and dependents is cued here not by length, as in the main text experiment, but only by the composition of the strings. Heads and dependents are equal in length. Note that heads were changed so that the number of letters in all strings was the same. The full set of heads and dependents is shown in Table 4.

**Table 4.** Non-word head and dependent stimuli (equal length).

Heads		Dependents	
Head1	nageng, negang,	Dep1a	bavbav, bazbaz, davdav, dazdaz
	genang, ganeng	Dep1b	vebveb, vedved, zebzeb, zedzed
Head2	rukork, rokurk,	Dep2a	pufpuf, puspus, tuftuf, tustus
	kurork, korurk	Dep2b	fopfop, fotfot, sopsop, sotsot

#### Shape stimuli

Stimuli were comprised of shapes. Heads and dependents were the same shapes as in the experiment reported in the main text, and all cues to categories remain the same, expect in this case, heads and dependents were the same size. The difference between heads and dependents here is, therefore, simply the general composition of the shapes. The full set of heads and dependents is shown in Table 5.

**Table 5.** Shape head and dependent stimuli (equal size).

Heads		Dependents	
Head1	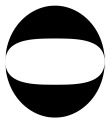 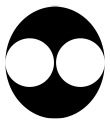 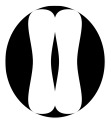 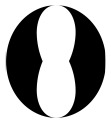	Dep1a	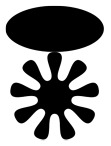 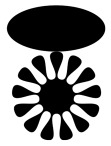 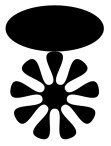 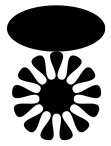
		Dep1b	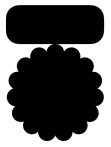 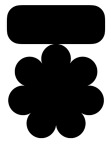 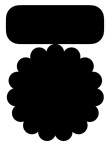 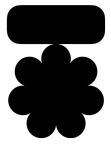
Head2	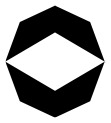 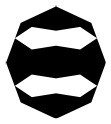 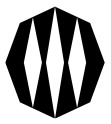 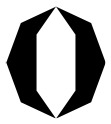	Dep2a	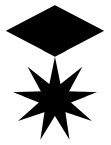 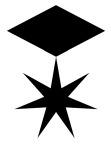 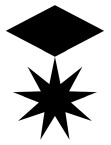 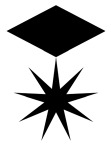
		Dep2b	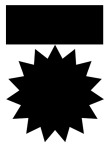 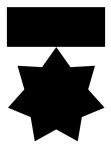 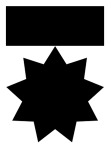 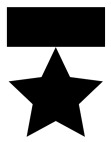

### Procedure

The procedure was identical to Experiments 1–4 above.

### Results

**Figure 5.**
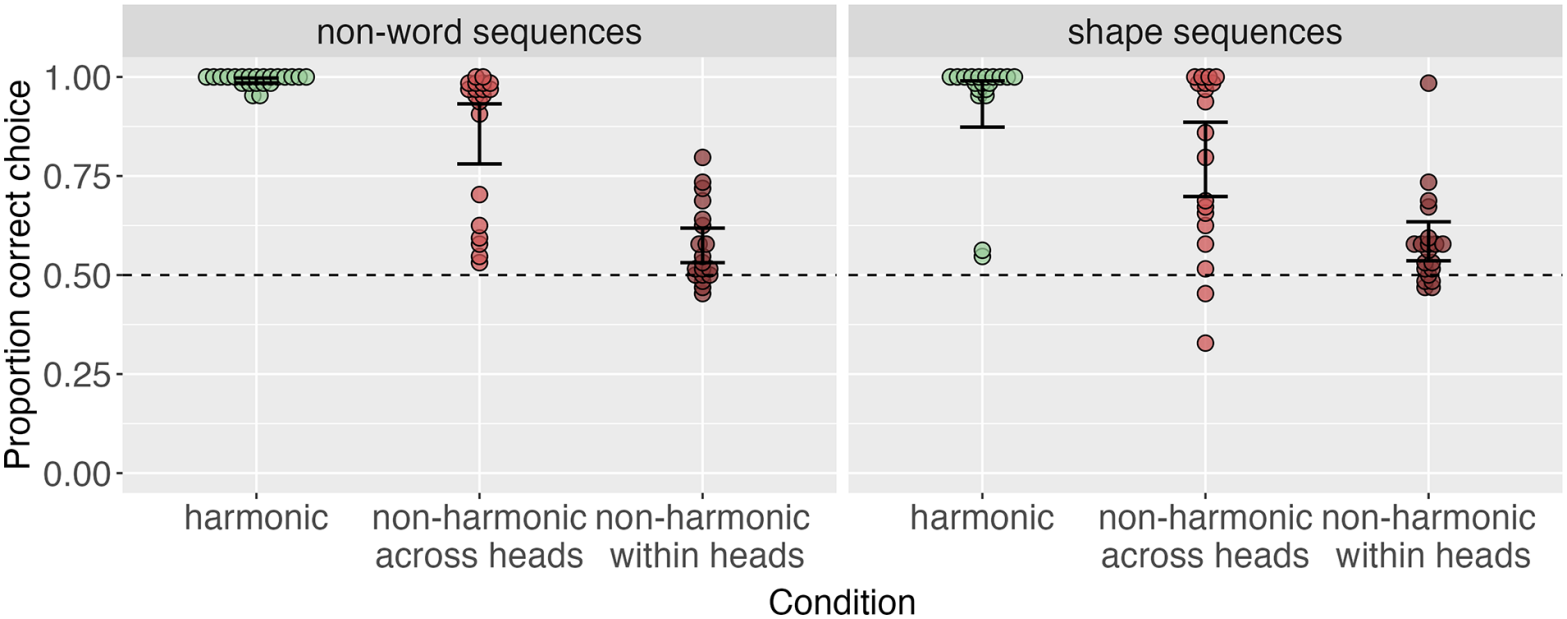
Order accuracy across conditions in each stimulus domain in a replication where heads and dependents are equal length/size. As in the main text experiments, harmonic conditions are learned with the highest accuracy; non-harmonic within heads conditions are learned with the lowest accuracy.

Figure 5 shows accuracy in learning order by condition for non-word and shape sequences. The patterns of results are the same as in the main text experiments. A model predicting order accuracy by condition (Helmert coded, with harmonic as the baseline), stimulus domain (treatment coded with shapes as the baseline), and their interaction revealed no significant main effect of experiment $\left(\chi^{2}(1)=1.25,p=0.26\right)$ , a main effect of condition $\left(\chi^{2}(1)=106.67,p<0.001\right)$ ; harmonic vs. non-harmonic: β=−1.27±0.20,p<0.001 ; non-harmonic across vs. within: β=−1.09±0.10,p<0.001) , and no significant interaction $\left(\chi^{2}(2)=3.79,p=0.15\right)$ .

### Discussion

Here, we tested whether changing the characteristic features that distinguished heads and dependents changes the pattern of preference for harmonic over non-harmonic order. We found that it did not. This finding supports our main claim that the preference for harmony in language reflects a domain-general bias for simplicity in learning. The simplicity bias as we have described it here relates directly to the notion of compressibility: more compressible rules are easier to learn (Chater & Vitányi, 2003). The compressibility of ordering rules is importantly independent of what defines the categories to be aligned. Easy-to-learn or highly similar categories may facilitate rule learning in general, but it should not change the advantage that harmony has over disharmony. Below we discuss the general implications of these results for the relationship between harmony and the notions of similarity that drive linguistic categories to align.

## General discussion

Across languages, some types of grammatical patterns seem to be more common than others. The history of linguistics is, in a sense, a history of explanations for such observations. Further, the sorts of explanations posited reflect deep debates about what drives languages to be similar to one another and to vary. Here, we have focused on one very well-known case of apparent similarity between languages: the tendency for certain categories of words to align with one another. Languages where verbs come before objects tend to have adpositions before nouns, and languages where verbs come after objects tend to have adpositions after nouns. Similarly, languages where adjectives come before nouns also have numerals and demonstratives that come before nouns; if adjectives instead follow nouns, then so will demonstratives and adjectives. While this tendency, known as word order harmony, has been extensively documented in languages (Dryer, 1992a; Dryer and Haspelmath, 2013; Greenberg, 1963; Liu, 2010), the ultimate explanation for it is less clear. Indeed, the kinds of alternative explanations proposed for word order harmony reflect different views about the role of human cognition in shaping why languages look the way they do. On the one hand, word order harmony might reflect, to some degree, accidents of history: certain categories of words, like verbs and adpositions, often share a historical source and, thus, by default, share their word order. In contrast to this, word order harmony has also been proposed to result from domain-specific representations and mechanisms: a single underlying structure used to derive all types of phrases, with language learners inferring one of two versions, syntactic heads first or last. Here, we explored a third possibility: that the source of harmony lies outside the language faculty in a domain-general bias for simplicity. This bias would operate on the linearization of language-specific categories and representations.

We explored this using a series of pattern-learning experiments. In each experiment, we compared learning of harmony-like patterns to learning of two kinds of increasingly complex non-harmonic patterns. We tested the prediction that, across non-word, shape, auditory, and tactile sequence learning, participants would find harmonic patterns easiest to learn, and non-harmonic patterns harder to learn. We predicted that the most complex type of non-harmonic pattern—where orders *within* each category of head-like elements could vary—would be the hardest to learn. These predictions were borne out, with similar patterns of learning across each stimulus type. This held even though the overall difficulty of learning differed across modalities. Further, our results held even when we changed the specific features that distinguished heads from dependents in our stimuli. While, in principle, other domains and particular stimulus categories could be tested, our results suggest these patterns of results would likely generalize.

While we have focused on harmonic vs. non-harmonic patterns in motivating our work, the two non-harmonic conditions in our experiments also help to illustrate the relationship between harmony and simplicity (or compressibility). In the *harmonic* conditions, participants can, in principle, learn the ordering pattern without separately learning the order for each class of heads and dependents—for example, by simply noticing that heads come first or last. This is precisely what makes harmonic patterns simpler. Note that the dependency results show that participants aren’t *only* learning this fact. They do learn the category structure of the patterns to which they are exposed. Nevertheless, the simpler, harmonic ordering pattern is easier to learn. In the *non-harmonic across heads* conditions, participants must make use of the different classes in order to learn the ordering pattern. They need to learn one generalization (or rule) for one class of heads and a different one for the other. Finally, in the *non-harmonic within heads* conditions, the ordering of dependent subclasses must also be learned since these determine the very specific ordering patterns for particular head and dependent combinations. In other words, the more information that is relevant to learning a particular pattern, the more complex it is.

These results point to a cognitive bias for patterns that resemble word order harmony in a key respect: they involve consistent linear alignment across distinct instances of elements that are similar along a salient dimension. It is worth revisiting two important aspects of our hypothesis in light of the results we have reported here. First, how do the categories—that is, the notions of similarity across items—that participants learn in our experiments relate to natural language categories implicated in word order harmony? Second, how can learning biases on the part of individuals shape population-level (i.e., cross-linguistic) tendencies? As discussed above, the harmonic alignment of specific linguistic categories has been argued to be determined by a number of distinct notions of similarity. These include the seemingly very abstract notions of head and dependent. In some syntactic theories, heads and dependents each occupy a distinct structural position in a hierarchy that is reused as the representational basis of every type of phrase (Baker, 2001; Chomsky, 1970, 1988; Travis, 1984). Harmony is about how this structure is linearised (i.e., it can be considered distinct from the hierarchy itself; Abels & Neeleman, 2012). Heads with a consistent linear order (first or last) relative to their dependents are harmonic. However, while this structure itself is widely assumed in at least some linguistic frameworks, which elements are heads is not always agreed upon. For example, while verbs and adpositions are generally treated as heads, in the noun phrase, the precise structure is contested. Under some approaches, and in some languages, the noun is the head (Jackendoff et al., 1977; Svenonius, 1994; Valois, 1991); in others, certain modifiers, like adjectives, are themselves treated as heads (Abney, 1987; Delsing, 1993; Truswell, 2006). In addition to a lack of consensus in some cases regarding which grammatical categories are heads, alternative theories of harmony have suggested that head vs. dependent is *not* what determines alignment. These alternatives point to less abstract dimensions of similarity, including whether a phrase is likely to consist of a single word or not (Dryer, 2009), or the degree to which two grammatical categories are semantically or functionally similar (Croft, 2002; Dryer, 1992b). Interestingly, on these measures, verbs and adpositions are predicted to align, but verbs and adjectives are not. Support for this comes from the fact that the latter shows no robust pattern of harmony cross-linguistically (Dryer, 1992a, 2009). These less abstract notions of similarity also directly relate to the likelihood with which two categories share a historical source—an alternative theory of harmony that is typically assumed to be noncognitive and unrelated to any pressure for consistent order. Under this theory, verbs and adpositions align because they share a historical source. However, as Wang et al. (2024) argue, experimental evidence connecting semantic similarity to harmony in fact suggests that pathways of change and harmony may be related. Specifically, they may both be driven by a general mechanism for analogical mapping, based on similarity (e.g., Gentner & Kurtz, 2006; Russell, 1988). On the one hand, similarity may increase the likelihood that two elements will be structurally aligned to create a more compressible representation of the system (Culbertson & Kirby, 2016). On the other hand, similarity may explain why certain classes of words are regularly recruited as sources for others diachronically. These processes may feed each: sharing a historical source may encourage harmony (Dryer, 1992b), and harmony between two categories may increase their similarity (Gentner & Maravilla, 2017). Importantly, this view also supports our claim that the dimensions of similarity that drive harmony may be in part specific to language, but they are not radically different from the features of similarity that are relevant in our stimuli. What is crucial for us is that generalisation of rules from one pair of elements to another is sensitive to the similarity in general.

Turning to the second question, often called the problem of linkage (Kirby, 1999), how does the cross-linguistic tendency for harmony actually arise from individual-level learning biases. One highly general answer to this question is that the biases of individual learners—even relatively weak ones—can be amplified over time through cultural transmission (i.e., iterated learning Kirby et al., 2015; Thompson et al., 2016). In other words, when linguistic systems are passed from one generation to the next, the biases compound at each generation. While in principle this might lead to a stable distribution of languages in which harmony holds across the board (Kalish, Griffiths, & Lewandowsky, 2007), as shown in Figure 1, this is certainly not the case in the current distribution. Therefore, it is worth considering why the learning bias for harmony would *not* lead all languages to harmonise all phrases. While the reasons for this are likely complex, it should be clear from the preceding discussion that not all grammatical categories are equally similar to each other. Differences in similarity imply that the pressure to harmonise will not be equally present across all phrase types. Where similarity does not drive harmony, other potentially competing forces will determine order. If we assume that the dynamics of language change are such that eventually any type of language could potentially change into any other type given enough time, then over a potentially very long period of time we can predict that one type of perfectly harmonic language (e.g., one with all heads first) will eventually change into another type of harmonic language (e.g., one with all heads last). However, because language change is incremental, the pathway of change from one type to the other will pass through non-harmonic states where word orders are mixed. Crucially, the probabilities of transitioning between states at any one point in time are non-equal. Harmonic states will be more stable, and non-harmonic ones less so. The outcome of this set of dynamics is one where there is, at any one point in time, a mix of harmonic and non-harmonic languages, but the harmonic ones predominate (Kirby, 1999). To summarise, individual-level learning biases can lead to population-level cross-linguistic tendencies through cross-generational transmission. At the same time, achieving simplicity (or compressibility) by word order alignment necessarily depends on having a basis for alignment between categories, and just like in our stimuli, similarity is not uniform among linguistic categories. On top of that, harmonic and non-harmonic patterns evolve during language change in a stepwise fashion that makes transitions among harmonic patterns rare but does not rule them out entirely.

Returning to the implications of our findings for harmony, the results of our experiments demonstrate that perhaps the most well-attested organising principle governing cross-linguistic variation in word order may arise from a domain-general cognitive bias for simplicity. These results add to the growing body of evidence linking learners’ behaviour in artificial language and pattern learning experiments to constraints on variation in the world’s languages (Culbertson and Adger, 2014; Fedzechkina et al., 2012; Maldonado and Culbertson, 2020; Martin and White, 2019). Importantly, in this case, we have evidence that the bias driving alignment between categories does not depend on domain-specific mechanisms: the effects of this simplicity bias on sequential ordering replicate with striking similarity across multiple modalities and domains. Importantly though, this domain-general bias has domain-specific effects. Harmony in language is sensitive to categories, such as noun, adposition, and so on, that are linguistic in nature. Indeed, the specific categories that align with one another are the subject of years of debate in linguistics (Dryer, 2009; Dunn et al. 2011; Hawkins, 1994; Travis, 1984), and pattern-learning experiments can, in fact, shed light on what dimensions of similarity are relevant for the formation of compressible rules in language (e.g., see Wang et al., 2024). This type of explanation for patterns of cross-linguistic variation—whereby domain-general cognitive biases interact with domain-specific categories and representations—may have widespread applicability (Culbertson and Kirby, 2016). Carefully delineating the domain-general causes and the domain-specific effects underpinning cross-cultural patterns of human behaviour may be the best strategy for revealing the organising principles of human cognition.

## Notes

1. Indeed, identifying a learning set of categories proved more difficult in the initial piloting of auditory and tactile conditions. While it is difficult to assess how people perceive relative similarity across these modalities, an anonymous reviewer points out that string-based edit distance between heads and dependents may differ across our stimulus domains. For example, treating each head-dependent combination as a string (and ignoring any other dimensions of similarity), length-normalised edit distance between nageng and bav is 0.833, while between ._ _ _ _ . and _ . _ . it is 0.5. This could have made heads and dependents in the audio/tactile stimuli more difficult for participants to distinguish.
